# Credibility of Scientists: Conflict of Interest and Bias

**DOI:** 10.1289/ehp.114-a147b

**Published:** 2006-03

**Authors:** Jennifer Sass

**Affiliations:** Natural Resources Defense Council, Washington, DC, E-mail: jsass@nrdc.org

In their commentary, Barrow and Conrad (2006), both employed by the chemical industry, argued that industry-funded science and scientists are high quality and unbiased, and this is enforced through policies and practices such as disclosure of funding sources in scientific journals, guidelines for Good Laboratory Practices, peer review, the scientific process of independent repeatability, various federal laws, and the prospect of tort liability. Ironically, these same mechanisms have publicly revealed the often successful efforts by industry to weaken the regulation of their products.

The current checks and balances cited by Barrow and Conrad (2006) are not always effective guards against biased or even bad science. Numerous examples of biased industry science have been reported in the scientific literature:

- In an article co-authored by U.S. Environmental Protection Agency (EPA) scientists, Dearfield et al. (1993) compared the results from registrant-submitted mutagenicity studies to the U.S. EPA Office of Pesticide Programs with those from the published literature. The authors reported a selection bias, in which registrant-submitted studies on atrazine mutagenicity were all negative (no mutagenic activity), whereas over a dozen studies in the published literature reported mutagenic activity.
- In an analysis of studies submitted to the U.S. EPA on the effects of atrazine on frog reproductive development, Hayes (2004) reported that financial sponsorship was a strong predictor of study outcome (*p* = 0.009). Funding sources varied for studies reporting adverse effects (including government and industry funding), whereas all of the studies that failed to detect adverse effects were funded by the manufacturer of atrazine.
- In an analysis of 115 published studies on low-dose effects of the plastics-component bisphenol A, vom Saal and Hughes (2005) reported that > 90% of government-funded studies found significant low-dose effects, whereas none of the industry-funded studies did. More specifically, the authors found that
  Some industry-funded studies have ignored the results of positive controls, and many studies reporting no significant effects used a strain of rat that is inappropriate for the study of estrogenic responses. (vom Saal and Hughes 2005)
- Studies of documents from the tobacco industry archives have revealed evidence of concerted industry efforts to obscure the contribution of secondhand smoke and other environmental toxics to disease through the development of their own version of “good epidemiological practices” and “sound science” (Ong and Glantz 2001).

As Barrow and Conrad (2006) pointed out, federal scientific advisory committees and the National Academies want to include relevant experts, and therefore may appoint industry experts despite direct financial conflicts. As a solution, the International Agency for Research on Cancer (IARC) sometimes invites financially conflicted experts to speak to the committee but bars them from drafting documents or voting on evaluations (Cogliano et al. 2004). Prompt implementation of strict conflict guidelines (similar to those adopted by IARC) by the U.S. government and the National Academies should be a high priority. An editorial in The *Lancet* 2002 warned,

Members of expert panels need to be impartial and credible, and free of partisan conflicts of interest, especially in industry links or in right-wing or religious ideology.

Barrow and Conrad (2006) argued that I am biased because my work on scientific integrity is funded by a private foundation. However, there is no financial stake in the regulation of toxics for myself, my employer, or my funders. Moreover, the funders do not review or comment on my prepublication work or influence my work product in any way. I consistently acknowledge a bias toward ensuring that regulations of toxic chemicals are as health protective as feasible, consistent with the U.S. EPA’s stated goal—“to protect human health and the environment” (U.S. EPA 2005).
